# Prediction of the Quality of Anxi Tieguanyin Based on Hyperspectral Detection Technology

**DOI:** 10.3390/foods13244126

**Published:** 2024-12-20

**Authors:** Tao Wang, Yongkuai Chen, Yuyan Huang, Chengxu Zheng, Shuilan Liao, Liangde Xiao, Jian Zhao

**Affiliations:** 1 Institute of Digital Agriculture, Fujian Academy of Agricultural Sciences, Fuzhou 350003, China; solow2024@163.com (T.W.); stonecyk@126.com (Y.C.);; 2 Fujian Zhi Cha Intelligent Technology Co., Quanzhou 362400, China

**Keywords:** Tieguanyin, hyperspectral imaging, chemometrics, preprocessing, content prediction

## Abstract

Anxi Tieguanyin belongs to the oolong tea category and is one of the top ten most famous teas in China. In this study, hyperspectral imaging (HSI) technology was combined with chemometric methods to achieve the rapid determination of free amino acid and tea polyphenol contents in Tieguanyin tea. Here, the spectral data of Tieguanyin tea samples of four quality grades were obtained via visible near-infrared hyperspectroscopy in the range of 400–1000 nm, and the free amino acid and tea polyphenol contents of the samples were detected. First derivative (1D), normalization (Nor), and Savitzky–Golay (SG) smoothing were utilized to preprocess the original spectrum. The characteristic wavelengths were extracted via principal component analysis (PCA), competitive adaptive reweighted sampling (CARS), and the successive projection algorithm (SPA). The contents of free amino acid and tea polyphenol in Tieguanyin tea were predicted by the back propagation (BP) neural network, partial least squares regression (PLSR), random forest (RF), and support vector machine (SVM). The results revealed that the free amino acid content of the clear-flavoured Tieguanyin was greater than that of the strong-flavoured type, that the tea polyphenol content of the strong-flavoured Tieguanyin was greater than that of the clear-flavoured type, and that the content of the first-grade product was greater than that of the second-grade product. The 1D preprocessing improved the resolution and sensitivity of the spectra. When using CARS, the number of wavelengths for free amino acids and tea polyphenols was reduced to 50 and 70, respectively. The combination of 1D and CARS is conducive to improving the accuracy of late modelling. The 1D-CARS-RF model had the highest accuracy in predicting the free amino acid (R_P_^2^ = 0.940, RMSEP = 0.032, and RPD = 4.446) and tea polyphenol contents (R_P_^2^ = 0.938, RMSEP = 0.334, and RPD = 4.474). The use of hyperspectral imaging combined with multiple algorithms can be used to achieve the fast and non-destructive prediction of free amino acid and tea polyphenol contents in Tieguanyin tea.

## 1. Introduction

Tea is one of the most consumed beverages in the world [1], and it is rich in polyphenols, free amino acids, and other active ingredients, which have been shown to have a wide range of biological and pharmacological benefits, including antioxidant, hypolipidaemic, and anticancer effects [2]. According to the China Tea Consumption Market Report 2021, oolong tea production was 287,200 tons, with an increase of 9400 tons over the previous year [3]. As a typical “semifermented” tea, oolong tea is known for its elegance, floral aroma, and rich flavour. Among all types of tea, oolong tea requires the most complicated processing technology [4]. Oolong tea is an excellent quality tea produced through killing, withering, shaking, fermentation, baking, and other processes. Oolong tea is characterized by its sturdy and tight shape and its brownish and oily colour [5]. Oolong tea is one of the most popular teas in China, with Anxi Tieguanyin being the most famous, best, and most expensive oolong tea on the market [6]. Anxi Tieguanyin can also be categorized into two types, namely, the clear-flavoured type and the strong-flavoured type, according to the different processing techniques used. Teas of different grades differ in terms of colour, taste, and quality. The differences between these grades are significant, and the grade and contents of discriminatory identifiers of tea have important value and significance.

Currently, the main methods of tea quality evaluation are traditional sensory evaluation, laboratory chemical testing methods, electronic tongues, and electronic nose technology, among which sensory evaluation is the only national standard for evaluating the grade of tea, though it is easily affected by the external environment and subjective factors, meaning this method lacks objectivity [7]. For laboratory chemical testing methods, including chemical reagent analysis and gas chromatography-mass spectrometry [8], although the results are accurate, the operation is cumbersome, the determination time is long, and the efficiency is low. Electronic tongues and electronic nose equipment, unlike traditional human sensory organs, can be used for aroma and taste detection to obtain data, but the cost is high, and because of the sensitivity of the sensor, the impact is greater [9]. In summary, the existing traditional detection methods have certain limitations, and determining how high-throughput rapid non-destructive testing can be achieved, whether in tea processing, finished product quality testing, or grading, will play an important role.

Hyperspectral imaging (HSI) is an emerging optical technology that provides rich images and spectral information [10] and has been widely used in various aspects of crop element uptake [11,12], pests and diseases [13,14,15], agricultural product processing [16,17], and food quality inspection [18,19] because of its advantages in terms of producing high-dimensional data. Moreover, HSI has attracted the attention of researchers in various fields and has great application prospects. To improve the accuracy of the spectral detection model, preprocessing the spectral data and screening the feature bands are necessary. The processing method used on the feature data is the main factor affecting the modelling results, and commonly used spectral data processing methods include first derivative (1D), second derivative (2D), Savitzky–Golay (SG) smoothing, multiple scattering correction (MSC), standard normal variation (SNV), and normalization (Nor). The results of many spectral experiments show that these methods can reduce the interference of external factors to a certain extent and improve model accuracy [20,21,22]. In addition, to obtain accurate spectral data, feature spectra can be screened to prevent the problems of more irrelevant information and high redundancy occurring in the full spectrum, save computational resources, and improve model performance [23,24]. Commonly used feature extraction approaches include the successive projection algorithm (SPA), principal component analysis (PCA), competitive adaptive reweighted sampling (CARS), and uninformative variable elimination (UVE) [25,26,27].

Current spectral detection technology is applied mainly to black tea, dark tea, and green tea, as well as the quality detection of fresh tea leaves [28,29,30,31], which mainly detects aspects such as the tea chlorophyll content, tea polyphenol content, and free amino acid content [32,33]. However, research on oolong tea has focused on varietal differentiation and the identification of adulteration [34,35], and there are fewer discriminative identifications for the different types and grades of Anxi Tieguanyin. In addition, the effects of different band selection approaches and modelling algorithms on the performance of the prediction model for the compositional content of the finished tea of Anxi Tieguanyin have rarely been reported.

In this study, a HSI system was used to collect hyperspectral data on Anxi Tieguanyin clear-flavoured and strong-flavoured national standard first- and second-grade teas and to determine the content of tea polyphenols and free amino acids in each sample. The 1D, Nor, and SG smoothing preprocessing methods were used to eliminate the interfering background information, and the CARS algorithm, SPA algorithm, and PCA algorithm were used to extract the characteristic wavelengths. Four different prediction models—namely, a back propagation (BP) neural network, partial least squares regression (PLSR), a random forest (RF), and a support vector machine (SVM)—were established. The performance of these four models was compared and evaluated, from which the best model for predicting the quality of Anxi Tieguanyin tea polyphenols and free amino acids was selected. The results of this study provide a theoretical basis for non-destructive testing of Anxi Tieguanyin tea quality. In addition, the samples used in this study are national standard samples that can fully represent the different categories and grades of Anxi Tieguanyin, which is highly practical.

## 2. Materials and Methods

### 2.1. Experimental Materials

**Figure 1:**
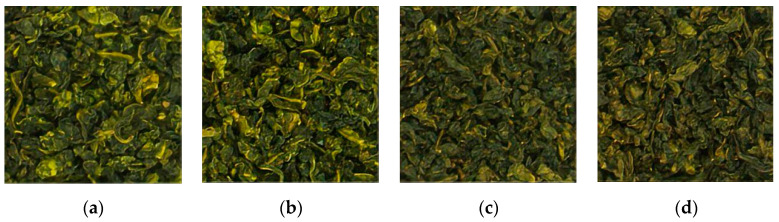
Samples of different grades of Tieguanyin tea leaves. (**a**) Clear-flavoured grade 1 (strong, firm, green oil, sand green, bright, uniform, and clean); (**b**) Clear-flavoured grade 2 (curly, firm, oily green, sandy green, still well-balanced, still clean, and slightly tender stalks); (**c**) Strong-flavoured grade 1 (fatter, firmer, oily, sandy green and brighter, even, and clean); (**d**) Strong-flavoured grade 2 (slightly plump, slightly firm, dark green, sandy green, still well-balanced, still clean, and young, slightly tender stalks).

The Tieguanyin tea samples selected for the experiments were provided by Yunling Tea Co. The samples were produced and labelled into 4 different grades in strict accordance with the national standard of GB/T19598-2006 [36]: namely, clear-flavoured grade 1, clear-flavoured grade 2, strong-flavoured grade 1, and strong-flavoured grade 2. The tea leaves of the four quality grades were weighed via an electronic balance, and 40 samples of each quality grade were collected. A total of 160 samples, each weighing 5 g ± 0.005 g, were encapsulated in individual bags, and all the samples were stored at 20 °C in a room-temperature environment. HSI acquisition was subsequently performed. The tea samples are shown in Figure 1.

### 2.2. HSI Data Acquisition

**Figure 2:**
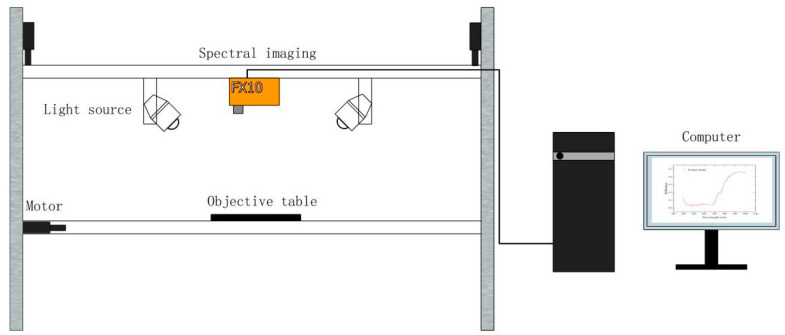
Schematic diagram of hyperspectral acquisition device.

A visible near-infrared HSI system (FX10, SPECIM, Oulu, Finland) was used to collect HSI data from the Tieguanyin tea samples. The system consists of a light source, a hyperspectral imager, a lens, an electronically controlled displacement platform and a computer (shown in Figure 2). The light sources used in the study were six 50 W tungsten-halogen lamps (GU5.3, PHILIPS, Suzhou, China). The wavelength range used was 300–2500 nm, the colour temperature was around 3100 K, and the guaranteed lamp lifetime was 2000 h. The hyperspectral camera had a spectral scanning range of 400–1000 nm, a spectral band of 224, a spectral resolution of 5.5 nm, a slit width of 30 μm, a spatial pixel count of 1024, a pixel size of 8 × 8 μm, and an acquisition speed of up to 327 Hz across the spectrum.

Prior to the acquisition of the HSI data, the system was warmed for 30 min to ensure its stability. The moving speed of the electronically controlled displacement stage was 2 mm/s, the exposure time was 10 ms, and the vertical distance between the hyperspectral camera and the samples was 40 cm. The tea samples were uniformly laid flat in a black vessel with a diameter of 9.3 cm and a height of 2.9 cm for hyperspectral scanning.

The process of acquiring sample data via an HSI system is affected by the uneven intensity distribution of the light source in all bands and the presence of dark current noise, which causes the quality of the acquired hyperspectral images to degrade. To reduce noise and correct the image, the original hyperspectral data are corrected with a standard whiteboard (an orbicular white board composed of polytetrafluoroethylene material) [37]. The correction formula is shown in Equation (1):

(1) $$R=\frac{R_{raw}-R_{dark}}{R_{white}-R_{dark}}$$

where R is the corrected spectral image, $R_{raw}$ denotes the original hyperspectral image, $R_{white}$ is the whiteboard image captured with the light turned on, and $R_{dark}$ is the all-black image when the lens is turned off.

### 2.3. Determination of the Contents of Free Amino Acid and Tea Polyphenol

Free amino acid was determined with reference to the national standard GB/T 8314-2013 [38] via the ninhydrin colorimetric method. A total of 0.3 g of tea powder was placed in a 100 mL conical flask, to which 45 mL of distilled water that had been boiled was added, and the mixture was then placed in a boiling water bath for 45 min. Next, the mixture was filtered immediately while it was hot, and the filtrate was transferred to a 50 mL volumetric flask; the volume was then fixed, and the mixture was shaken well. Two millilitres of tea broth was removed, and 0.5 mL of phosphate buffer (pH 8.0) and 2% ninhydrin solution (0.5 mL) was added. The mixture was heated in a boiling water bath for 15 min, cooled, and then brought to 25 mL, after which it was shaken well. The reagent blank mixture was used as a control, and the absorbance value was determined at a wavelength of 570 nm.

Tea polyphenols were determined with reference to the national standard GB/T 8313-2018 [39] via the Folin-Ciocalteu colorimetric method. A total of 0.2 g of tea powder was placed in a 10 mL centrifuge tube, 5 mL of 70% methanol solution was added at 70 °C, and the mixture was extracted for 10 min in a water bath at 70 °C. After the extraction had been repeated 2 times, the filtrate was combined and fixed in a 10 mL volumetric flask. One millilitre of tea broth was removed, and five millilitres of Folin-Ciocalteu mixture was added and shaken. The mixture was incubated for 5 min, 4 mL of sodium carbonate solution was added, the reagent blank solution was used as the control, and the absorbance value at a wavelength of 765 nm was determined.

### 2.4. Spectral Preprocessing Algorithm

ENVI5.3 software was used to delineate the region of interest (ROI) of the same location size, extract the spectral values of all the pixels of the ROI, and take the average of the region as the spectral value of the sample. The original dataset was processed in 1D [40]. By calculating the gradient between neighbouring wavelengths, the intervals with the most significant change in slope are observed, and the peaks of the first-order difference spectra are obtained from them. The larger peaks usually carry useful signal information, and it is also possible to determine the boundaries between the useful signal and the noise components on the basis of the locations of the peaks. Nor is used mainly to eliminate the effect of optical range differences on the spectral data. After the columns of the data are averaged, they are transformed to between 0 and 1, thus eliminating scale oversize, simplifying the data, and reducing the arithmetic. SG [41] smoothing utilizes polynomials for data smoothing based on the least squares method by fitting or averaging the data points within a certain window size around a single point of data (the width of the window is usually odd), analysing the signal by fitting or averaging the data points around a single point of data, and analysing the signal by a certain window size. Fitting or averaging is performed to analyse the useful information in the signal to estimate the ideal spectrum of the spectral data point, reduce the interference of the irregularly fluctuating noise signals in the spectral data on this data point, and improve the signal-to-noise ratio of the spectral data.

### 2.5. Feature Wavelength Extraction Algorithm

PCA [42] is an unsupervised learning algorithm, a technique for simplifying data sets, mainly used for downscaling and data compression. It can transform the relevant variables in the original dataset into a set of orthogonal principal components. These principal components are ranked in descending order according to their ability to explain the variance; this achieves the goal of preserving the main features of the data with fewer dataset dimensions. The basic idea of CARS variable screening [43] is to search for optimality on the basis of the screening of the partial least squares regression coefficients by adaptive reweighting, where wavelengths with larger weight assignments are selected, the wavelength points with smaller weight assignments are screened out, and the selected wavelengths are removed via the cross-validation method. The validation method is used to select the dataset with the smallest root mean square error of cross-validation (RMSECV). The optimal combination of wavelengths with the highest correlation to the labelled values can be selected via the above screening principle. SPA [43] uses the projection analysis of vectors to search for groups of variables that contain minimal redundant information in the spectral information, minimize the effect of covariance among variables, and reduce the overlap of information, while a few filtered out variables can represent most of the information of the original data. The speed and efficiency of modelling are thus improved.

With respect to dataset division, this study selected the training set and prediction set according to a ratio of 2:1 on the basis of the total number of samples.

### 2.6. Model Construction and Evaluation

PLSR [44] analyses mainly the regression modelling of multiple dependent variables or a single dependent variable on multiple independent variables, and the simplest form is the linear regression model between the dependent variable y and the independent variable x. RF [45] is a specific implementation of the bagging architecture in integrated algorithms, which involves constructing multiple CART decision trees in parallel and using the arithmetic average of the learning results of multiple decision trees as the regression results for the final model output. An SVM [46] is a supervised learning algorithm based on Vapnik-Chervonenkis dimension theory and the principle of structural risk minimization, which uses the limited information of training samples to seek the optimal balance between model complexity and learning ability. BP [47] is a multilayer feed-forward neural network based on the Winrow–Hoff learning rule and is widely used to simulate a variety of nonlinear relationships. The topology of the BP neural network includes input, hidden, and output layers, and the weights and biases of each layer are calculated by setting the network operation parameters (iteration number, learning rate, etc.). The weights and biases are continuously corrected via error inverse feedback via the gradient descent method, which ultimately minimizes the error sum of squares of the network.

The coefficient of determination (R^2^), root mean square error (RMSE), and relative percent deviation (RPD) were selected as the model evaluation indices. The performance of the built models was evaluated according to the determination coefficient of the training set (R_C_^2^) and test set (R_P_^2^), the root mean square error of the training set (RMSEC) and test set (RMSEP), and the relative deviation of the test set (RPD). In general, better models should have higher R_C_^2^, R_P_^2^, and RPD values and lower RMSEC and RMSEP values. In general, when the RPD value is less than 1.5, the constructed model has poor performance and cannot be used for predictive analysis. When the RPD value is between 1.5 and 2.0, the prediction effect of the constructed prediction model is average, and the sample can be roughly estimated. When the RPD value is greater than 2.0, the model has good predictive ability [37]. These indicators are calculated by the following formula:

(2) $$R^{2}=1-\frac{\sum_{i=1}^{n}{\left({\hat{y}}_{i}-y_{i}\right)}^{2}}{\sum_{i=1}^{n}{\left(y_{i}-\bar{y}\right)}^{2}}$$

(3) $$RMSE=\sqrt{\frac{\sum_{i=1}^{n}{\left(y_{i}-{\hat{y}}_{i}\right)}^{2}}{n}}$$

(4) $$RPD=\frac{SD}{RMSE}$$

where *n* is the number of samples, $y_{i}$ and ${\hat{y}}_{i}$ are the measured value and predicted value of the sample, $\bar{y}$ represents the average value of the sample, and *SD* is the standard deviation.

### 2.7. Data Analysis

**Figure 3:**
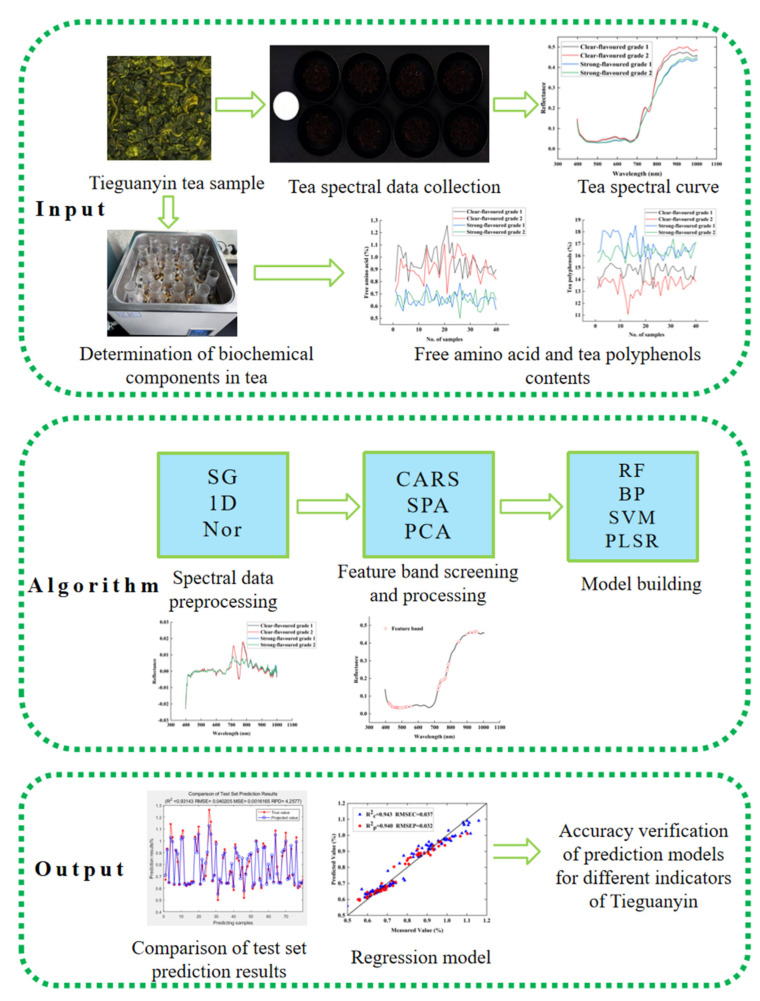
A flowchart of the methodology.

Figure 3 shows the flowchart of the methodology used in this work. The figures involved in the spectral analysis and modelling method were drawn using the following software: MATLAB R2020b and Origin 2021.

## 3. Results and Discussion

### 3.1. Statistical Analysis of the Biochemical Composition of Tieguanyin

As shown in Table 1, the free amino acid content of clear-flavoured Tieguanyin was greater than that of the strong-flavoured type, the free amino acid content of the first-grade clear-flavoured type was greater than that of the second-grade clear-flavoured type, the free amino acid content of the first-grade clear-flavoured type ranged from 0.80% to 1.26%, and the free amino acid content of the second-grade clear-flavoured type ranged from 0.70% to 1.11%. The difference in free amino acid content between the first-grade strong-flavour type and the second-grade strong-flavour type was not significant. In terms of tea polyphenols, the content of tea polyphenols in the strong-flavoured Tieguanyin group was greater than that in the clear-flavoured Tieguanyin group, and the contents in the first-grade flavoured Tieguanyin group were greater than those in the second-grade flavoured Tieguanyin group, with a small coefficient of variation.

### 3.2. Hyperspectral Data Preprocessing Analysis

**Figure 4:**
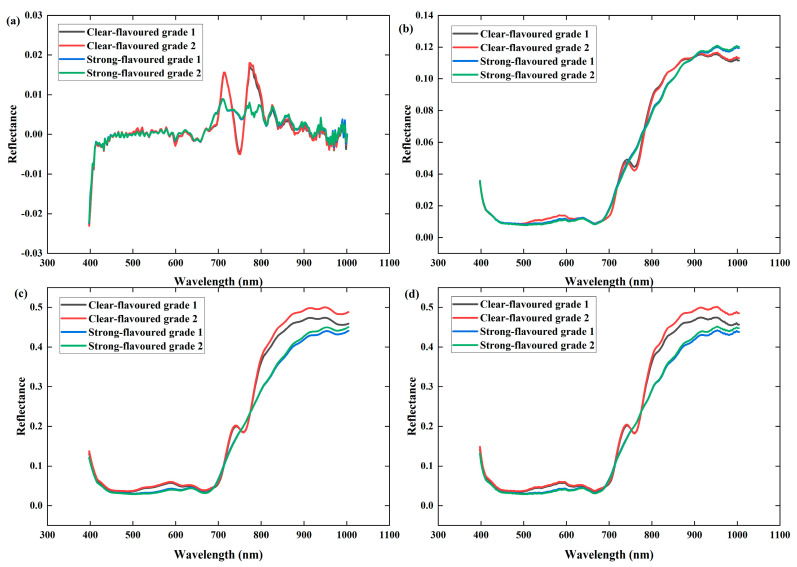
Original spectra and preprocessed spectra. (**a**) 1D preprocessing, (**b**) Nor preprocessing, (**c**) SG preprocessing, and (**d**) raw spectra.

To minimize the fluctuation of the spectral data and eliminate curve differences, different preprocessing methods were used for the raw spectra. The raw average reflectance spectrogram and the preprocessed spectral curve are shown in Figure 4. After 1D preprocessing (Figure 4a), the absorption peaks and reflection valleys of the spectra are clearly more prominent, which improves the sensitivity of the spectra and is conducive to subsequent analysis and identification. After the spectral curves were preprocessed with Nor (Figure 4b), the differences between the samples were reduced, and the smoothness was slightly improved. After the spectral curves were preprocessed by SG (Figure 4c), the shape and width of the spectral data were unchanged and smoother than those of the original spectral data because SG smoothing reduces noise and fluctuations in the data by locally fitting the spectral data and then replacing the original data points with the fitted values.

### 3.3. Feature Band Extraction Analysis

#### 3.3.1. PCA

**Figure 5:**
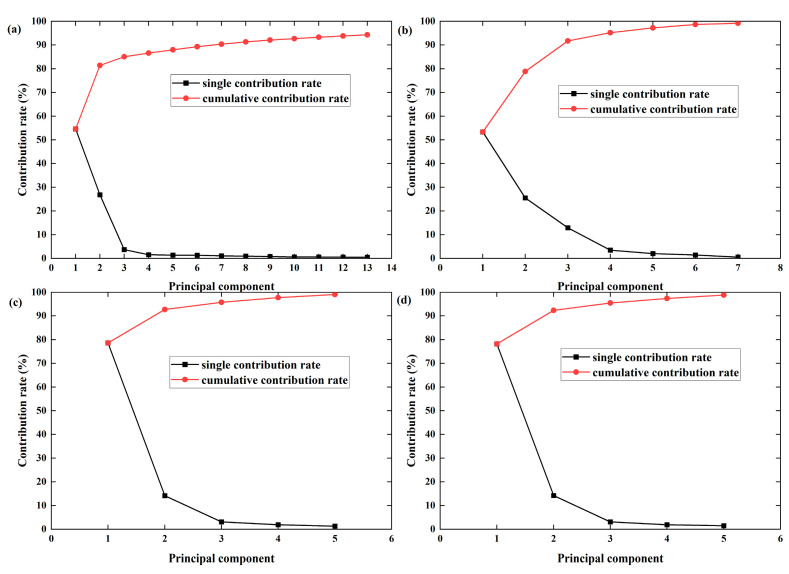
Contributions of components and cumulative contributions after PCA treatment. (**a**) 1D-preprocessed data, (**b**) Nor-preprocessed data, (**c**) SG-preprocessed data, (**d**) original spectrum.

The preprocessed spectral data and the original spectral data were downscaled via PCA, and the downscaling results are shown in Figure 5 (the black curves represent the individual component contribution rates, and the red curves reflect their cumulative contribution rates). Using PCA to extract the 1D-preprocessed feature data bands, the individual component contribution rates and cumulative contribution rates of the first thirteen principal components were obtained, as shown in Figure 5a. The cumulative contribution rate of the first seven principal components is greater than 90%, which indicates that the first seven principal components contain most of the effective information in the original spectral data. However, to save the more effective information and ensure the accuracy of the subsequent modelling, the principal components with eigenvalues greater than 1 were selected as the input data for constructing the model in the present study, and the cumulative contribution rate is 94.319% when the number of principal components is 13. Figure 5b shows the PCA extraction of the Nor-preprocessed feature data bands; when the number of principal components is seven, the cumulative contribution rate is 99.202%, i.e., the first seven principal components represent 99.202% of all the Nor data. Figure 5c shows the feature data band after PCA-extracted SG preprocessing, and the cumulative contribution rate is 99.094% when the number of principal components is five. Figure 5d shows the PCA-extracted feature data band of the original spectrum, and the cumulative contribution rate is 98.884% when the number of principal components is five.

#### 3.3.2. SPA Algorithm and CARS Algorithm for Extracting Feature Bands

**Figure 6:**
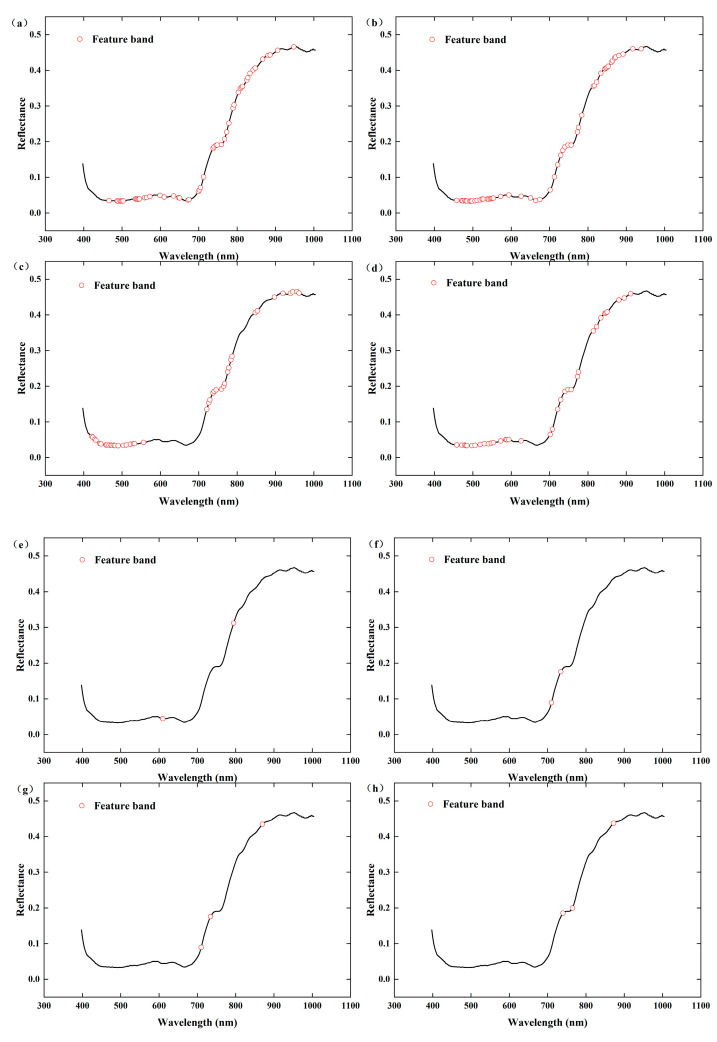
Feature bands of free amino acid screened by the CARS and SPA algorithms. (**a**) 1D-CARS, (**b**) Nor-CARS, (**c**) SG-CARS, (**d**) raw-CARS, (**e**) 1D-SPA, (**f**) Nor-SPA, (**g**) SG-SPA, and (**h**) raw-SPA.

**Figure 7:**
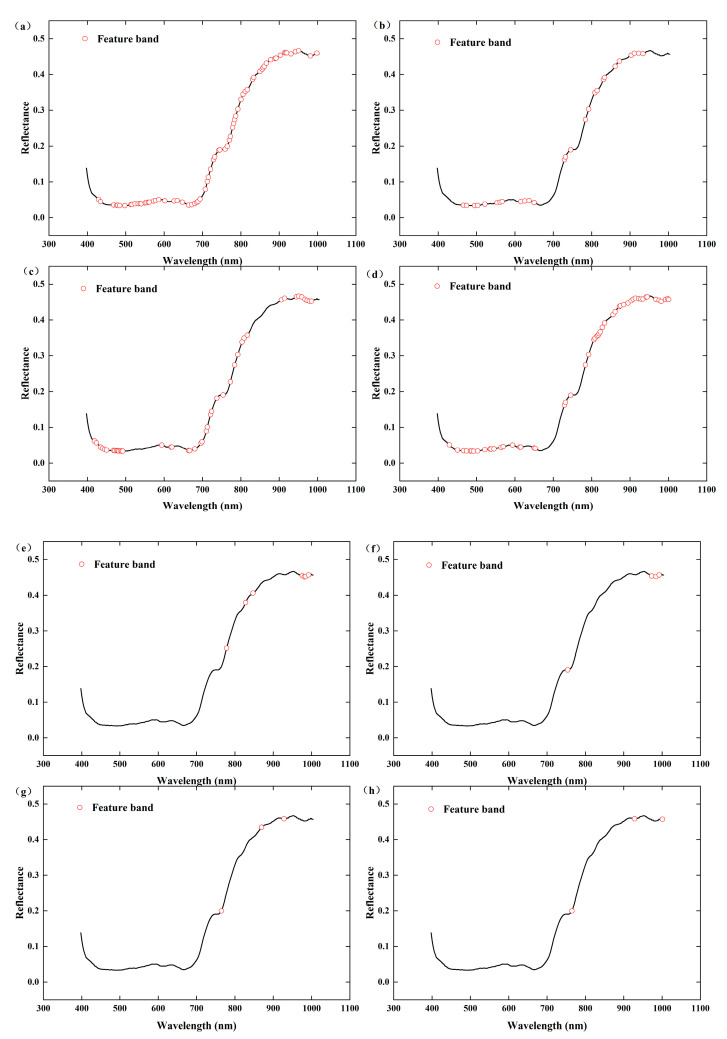
Feature bands of tea polyphenol screened by the CARS and SPA algorithms. (**a**) 1D-CARS, (**b**) Nor-CARS, (**c**) SG-CARS, (**d**) raw-CARS, (**e**) 1D-SPA, (**f**) Nor-SPA, (**g**) SG-SPA, and (**h**) raw-SPA.

To eliminate the influence of irrelevant wavelengths on model accuracy, feature bands were selected via the CARS and SPA algorithms, as shown in Table 2 and Figure 6 and Figure 7. Among the feature band selection methods related to free amino acids, the number of feature bands selected via the CARS algorithm after Nor preprocessing is the highest at 53, accounting for 23.66% of the total number of bands. Next, 1D preprocessing, which selects 50 bands, accounts for 22.32% of the total number of bands. The SPA algorithm selects the minimum number of feature bands, which is 2–3. Among the methods for selecting feature bands related to tea polyphenols, the number of feature bands selected via the CARS algorithm after 1D preprocessing is the highest at 70, accounting for 31.25% of the total number of bands. The SPA algorithm selects a relatively small number of feature bands, ranging from three to seven.

### 3.4. Comparative Analysis of Different Models

#### 3.4.1. Free Amino Acid Model Prediction Results

As shown in Table 3, among the four models for free amino acid prediction, the modelling effect of RF is better than that of the other three modelling methods. Compared with the direct use of the raw spectra, the RF model predictions are improved by using CARS feature wavelength extraction after processing with the 1D, Nor, and SG preprocessing algorithms. The correlation coefficients (R_C_^2^, R_P_^2^) of the RF model are greater than 0.9, the RMSEC and RMSEP are smaller, and the RPDs are greater than 2. The 1D-CARS-RF model has the best predictive performance, with an R_C_^2^ of 0.943 and an R_P_^2^ of 0.940. Additionally, the root mean square errors are minimized, with an RMSEC of 0.037 and an RMSEP of 0.032, and the RPD is 4.446. Among the different preprocessing prediction models that use the SPA feature wavelength extraction method, the correlation coefficients (R_C_^2^, R_P_^2^) and RPD values of SG-SPA-RF are higher than those of the other models at 0.912, 0.914, 3.848, respectively; meanwhile, the RMSEC and RMSEP are smaller than those of the other models at 0.044 and 0.040. Among the different preprocessing prediction models that use the PCA feature wavelength extraction method, the correlation coefficients (R_C_^2^, R_P_^2^) of the 1D-PCA-RF model are above 0.9, the RMSEC and RMSEP are smaller, and the RPD value is greater than 2, with values of 0.924, 0.929, 0.038, 0.039, and 4.454, respectively, which make the model predictions better.

The model that performs better with the BP modelling training set and test sets is Nor-CARS-BP, with an R_C_^2^ of 0.744, an R_P_^2^ of 0.738, an RMSEC of 0.078, an RMSEP of 0.084, and an RPD value of 2.032. The model that performs better with the PLSR modelling is raw-CARS-PLSR, with an R_C_^2^ of 0.896 and an R_P_^2^ of 0.847. When modelling with SVMs, with the exception of 1D-SPA-SVM, SG-SPA-SVM, and raw-SPA-SVM, the correlation coefficients (R_C_^2^, R_P_^2^) for the training and test sets of all other models are greater than 0.7.

#### 3.4.2. Tea Polyphenol Model Prediction Results

As shown in Table 4, among the four models for tea polyphenol prediction, RF modelling was better than the other three modelling methods. Compared with the direct use of the raw spectra, the prediction effect of RF modelling was improved after 1D, Nor, and SG preprocessing followed by CARS feature wavelength extraction. It is observed that 1D-CARS-RF has the best predictive performance. The training and test sets of 1D-CARS-RF have the highest correlation coefficients, with an R_C_^2^ of 0.939, an R_P_^2^ of 0.938, an RMSEC of 0.326, an RMSEP of 0.334, and an RPD of 4.474. Of the different preprocessing prediction models that use the SPA feature wavelength extraction method, the correlation coefficients (R_C_^2^, R_P_^2^) for the training and test sets of 1D-SPA-RF are 0.920 and 0.915, respectively, while the root mean square errors (RMSEC, RMSEP) for the training and test sets are 0.376 and 0.370, respectively; the RPD is 3.891, which indicates that the model performance is good. Of the different preprocessing prediction models that use the PCA feature wavelength extraction method, the R_C_^2^ and R_P_^2^ for the training and test sets of the 1D-PCA-RF model are greater than 0.9, the RMSEC and RMSEP values are smaller, the RPD value is greater than 2, and the model performs better regarding prediction.

The model with better performance in the training and test sets via BP modelling is Nor-CARS-BP, with an R_C_^2^ of 0.804 and an R_P_^2^ of 0.750. The model with the highest correlation coefficients (R_C_^2^, R_P_^2^) via PLSR modelling was raw-CARS-PLSR, with an R_C_^2^ of 0.911 and an R_P_^2^ of 0.819. The model that performs better with the SVM modelling training set and test sets was SG-CARS-SVM, with an R_C_^2^ of 0.789 and an R_P_^2^ of 0.776.

#### 3.4.3. Model Validation

**Figure 8:**
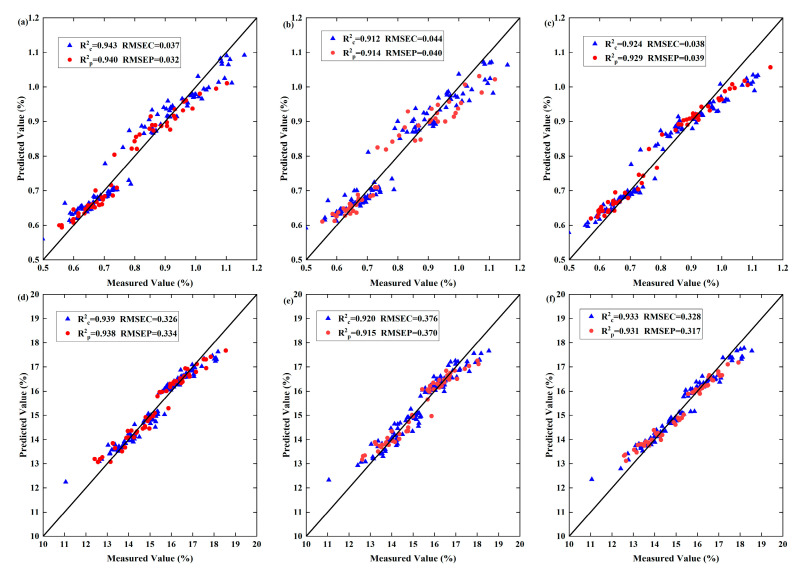
Scatterplot of the best model with different feature band extraction methods. (**a**–**c**) Free amino acid content prediction results obtained by the 1D-CARS-RF, SG-SPA-RF, and 1D-PCA-RF models; (**d**–**f**) tea polyphenol content prediction results obtained by the 1D-CARS-RF, 1D-SPA-RF, and 1D-PCA-RF models.

Through the above analysis, the optimal models for predicting the free amino acid and tea polyphenol contents can be filtered out (Figure 8). As the plots show, all six models have good predictive ability, and the measured and predicted values are very close to a 1:1 straight line, indicating that the predicted values of free amino acids and tea polyphenols in Tieguanyin have a good correlation with the measured values and that the established prediction models have good accuracy. The number of feature bands in the free amino acid prediction model 1D-CARS-RF was fifty, accounting for 22.32% of the total number of bands, and the number of feature bands in SG-SPA-RF was three (709.97 nm, 734.52 nm, and 869.47 nm), accounting for 1.34% of the total number of bands. The number of feature bands in the tea polyphenol prediction model 1D-CARS-RF was seventy, which accounted for 31.25% of the total number of bands, and the number of feature bands in 1D-SPA-RF was seven (778.34 nm, 827.92 nm, 847.29 nm, 975.65 nm, 981.27 nm, 984.09 nm, and 992.54 nm), which accounted for 3.13%. The extraction of feature wavelengths via 1D preprocessing combined with the CARS algorithm achieves the highest accuracy through RF modelling, whereas the extraction of feature wavelengths via the SPA algorithm not only simplifies the complexity of the free amino acid and tea polyphenol detection model of Tieguanyin tea but also reduces the modelling time.

## 4. Conclusions

In this study, HSI was combined with chemometrics: we collected hyperspectral data from four Tieguanyin tea samples of different quality grades and determined the free amino acid and tea polyphenol contents of each corresponding sample. We established and compared all the prediction models of different modelling approaches. This study demonstrates that the prediction accuracy of several models for assessing tea quality was significantly enhanced through the application of various preprocessing techniques and the careful selection of characteristic wavelengths. Among these models, the 1D-CARS-RF model exhibited superior performance, displaying high correlation coefficients and low prediction errors in predicting both free amino acid and tea polyphenol content. These findings strongly suggest the feasibility of employing hyperspectral technology for the non-destructive evaluation of Tieguanyin tea quality, providing a crucial theoretical foundation for the development of practical, non-invasive quality control methods.

## Figures and Tables

**Table 1:** Statistical analysis of the biochemical composition of Tieguanyin.

Biochemical Composition	Quality Level	Minimum	Maximum	Mean	Standard Deviation	Coefficient of Variation %
Free Amino Acid (%)	Clear-flavoured grade 1	0.80	1.26	0.97	0.10	10.70
	Clear-flavoured grade 2	0.70	1.11	0.91	0.10	11.00
	Strong-flavoured grade 1	0.56	0.79	0.66	0.05	8.19
	Strong-flavoured grade 2	0.50	0.74	0.66	0.06	8.39
Tea Polyphenol (%)	Clear-flavoured grade 1	13.21	15.87	14.72	0.61	4.12
	Clear-flavoured grade 2	11.06	14.50	13.52	0.67	4.98
	Strong-flavoured grade 1	15.45	18.55	16.82	0.85	5.05
	Strong-flavoured grade 2	15.35	17.42	16.27	0.47	2.87

**Table 2:** Feature band screening results.

Index	Screening Method	Preprocessing Method	Number of Bands
Free Amino Acid	CARS	1D	50
		Nor	53
		SG	41
		raw	35
	SPA	1D	2
		Nor	2
		SG	3
		raw	3
Tea Polyphenol	CARS	1D	70
		Nor	27
		SG	43
		raw	52
	SPA	1D	7
		Nor	4
		SG	3
		raw	3

**Table 3:** Free amino acid model prediction results.

Screening Method	Preprocessing Method	Model	Training Set		Test Set		
			R_C_^2^	RMSEC	R_P_^2^	RMSEP	RPD
CARS	1D	BP	0.799	0.076	0.651	0.093	1.823
		PLSR	0.756	0.073	0.716	0.080	2.081
		RF	0.943	0.037	0.940	0.032	4.446
		SVM	0.755	0.080	0.765	0.091	1.974
	Nor	BP	0.744	0.078	0.738	0.084	2.032
		PLSR	0.910	0.047	0.809	0.073	2.343
		RF	0.933	0.039	0.937	0.037	4.394
		SVM	0.771	0.081	0.738	0.084	1.933
	SG	BP	0.696	0.079	0.683	0.084	1.846
		PLSR	0.718	0.081	0.762	0.073	2.061
		RF	0.935	0.038	0.939	0.036	4.471
		SVM	0.751	0.085	0.753	0.076	2.013
	raw	BP	0.740	0.078	0.696	0.082	1.987
		PLSR	0.896	0.050	0.847	0.059	2.805
		RF	0.928	0.039	0.919	0.043	3.903
		SVM	0.748	0.082	0.766	0.094	1.957
SPA	1D	BP	0.699	0.088	0.499	0.113	1.294
		PLSR	0.234	0.116	0.561	0.091	1.513
		RF	0.858	0.054	0.866	0.051	3.152
		SVM	0.532	0.158	0.647	0.160	1.078
	Nor	BP	0.649	0.084	0.599	0.087	1.979
		PLSR	0.629	0.086	0.623	0.083	1.960
		RF	0.903	0.046	0.891	0.046	3.419
		SVM	0.740	0.162	0.723	0.183	1.033
	SG	BP	0.686	0.083	0.683	0.095	1.724
		PLSR	0.668	0.083	0.674	0.080	1.945
		RF	0.912	0.044	0.914	0.040	3.848
		SVM	0.640	0.115	0.677	0.122	1.450
	raw	BP	0.740	0.080	0.611	0.095	1.623
		PLSR	0.657	0.084	0.688	0.081	1.965
		RF	0.873	0.051	0.876	0.049	3.272
		SVM	0.655	0.112	0.654	0.111	1.541
PCA	1D	BP	0.770	0.078	0.708	0.087	1.896
		PLSR	0.710	0.081	0.717	0.083	1.852
		RF	0.924	0.038	0.929	0.039	4.454
		SVM	0.789	0.072	0.734	0.102	1.815
	Nor	BP	0.875	0.059	0.620	0.088	1.685
		PLSR	0.737	0.079	0.686	0.087	1.655
		RF	0.856	0.046	0.876	0.052	3.575
		SVM	0.772	0.084	0.728	0.084	1.889
	SG	BP	0.735	0.076	0.656	0.086	1.750
		PLSR	0.650	0.085	0.659	0.082	1.935
		RF	0.876	0.051	0.853	0.047	3.180
		SVM	0.748	0.090	0.726	0.076	1.900
	raw	BP	0.796	0.070	0.636	0.099	1.473
		PLSR	0.615	0.085	0.636	0.080	2.132
		RF	0.883	0.046	0.872	0.050	3.341
		SVM	0.746	0.086	0.716	0.092	1.811

**Table 4:** Tea polyphenol model prediction results.

Screening Method	Preprocessing Method	Model	Training Set		Test Set		
			R_C_^2^	RMSEC	R_P_^2^	RMSEP	RPD
CARS	1D	BP	0.736	0.674	0.782	0.657	2.013
		PLSR	0.803	0.619	0.742	0.712	1.831
		RF	0.939	0.326	0.938	0.334	4.474
		SVM	0.816	0.678	0.724	0.683	1.893
	Nor	BP	0.804	0.646	0.750	0.841	1.850
		PLSR	0.867	0.502	0.812	0.572	2.487
		RF	0.931	0.340	0.930	0.354	4.215
		SVM	0.751	0.742	0.720	0.793	1.872
	SG	BP	0.784	0.713	0.701	0.913	1.631
		PLSR	0.761	0.650	0.796	0.655	2.154
		RF	0.931	0.361	0.927	0.320	4.056
		SVM	0.789	0.665	0.776	0.762	2.029
	raw	BP	0.706	0.685	0.753	0.670	2.008
		PLSR	0.911	0.404	0.819	0.631	2.420
		RF	0.927	0.339	0.921	0.399	3.938
		SVM	0.769	0.683	0.756	0.826	1.892
SPA	1D	BP	0.749	0.647	0.627	0.876	1.690
		PLSR	0.705	0.704	0.730	0.678	2.142
		RF	0.920	0.376	0.915	0.370	3.891
		SVM	0.744	0.719	0.712	0.987	1.657
	Nor	BP	0.688	0.750	0.712	0.775	1.853
		PLSR	0.670	0.731	0.670	0.819	1.739
		RF	0.862	0.466	0.862	0.432	3.264
		SVM	0.712	0.879	0.684	0.950	1.598
	SG	BP	0.702	0.685	0.723	0.754	1.889
		PLSR	0.705	0.711	0.715	0.729	1.788
		RF	0.900	0.386	0.893	0.454	3.484
		SVM	0.777	0.794	0.714	0.882	1.673
	raw	BP	0.725	0.690	0.705	0.731	1.981
		PLSR	0.712	0.693	0.714	0.685	2.126
		RF	0.896	0.413	0.882	0.406	3.441
		SVM	0.751	0.877	0.755	0.972	1.564
PCA	1D	BP	0.696	0.763	0.623	0.766	1.791
		PLSR	0.748	0.694	0.696	0.690	1.805
		RF	0.933	0.328	0.931	0.317	4.483
		SVM	0.787	0.657	0.762	0.765	2.015
	Nor	BP	0.769	0.682	0.655	0.833	1.690
		PLSR	0.674	0.725	0.648	0.812	1.745
		RF	0.856	0.460	0.834	0.417	3.212
		SVM	0.769	0.755	0.726	0.741	1.904
	SG	BP	0.707	0.859	0.692	0.897	1.705
		PLSR	0.729	0.682	0.674	0.731	1.895
		RF	0.875	0.398	0.880	0.486	3.414
		SVM	0.779	0.709	0.759	0.722	2.000
	raw	BP	0.700	0.702	0.610	0.730	2.013
		PLSR	0.731	0.667	0.636	0.804	1.990
		RF	0.876	0.427	0.886	0.419	3.565
		SVM	0.778	0.699	0.766	0.750	2.011

## Data Availability

The original contributions presented in this study are included in the article. Further inquiries can be directed to the corresponding author.
